# Isolation of a Novel Bacterial Strain Capable of Producing Abundant Extracellular Membrane Vesicles Carrying a Single Major Cargo Protein and Analysis of Its Transport Mechanism

**DOI:** 10.3389/fmicb.2019.03001

**Published:** 2020-01-14

**Authors:** Chen Chen, Jun Kawamoto, Soichiro Kawai, Akihiro Tame, Chiaki Kato, Tomoya Imai, Tatsuo Kurihara

**Affiliations:** ^1^Institute for Chemical Research, Kyoto University, Uji, Japan; ^2^Marine Works Japan, Ltd., Yokosuka, Japan; ^3^Department of Marine Biodiversity Research, Japan Agency for Marine-Earth Science and Technology, Yokosuka, Japan; ^4^Research Institute for Sustainable Humanosphere, Kyoto University, Uji, Japan

**Keywords:** cold-adapted bacterium, extracellular membrane vesicles, protein secretion, *Shewanella*, type II protein secretion system

## Abstract

Extracellular membrane vesicles (EMVs) play an important role in various bacterial activities. EMVs have potential for use as vaccines, drug-delivery vehicles, platforms for extracellular production of recombinant proteins, and so on. In this study, we newly isolated a cold-adapted bacterium, *Shewanella vesiculosa* HM13, which abundantly produces EMVs, characterized them, and analyzed their cargo transport mechanism. *S. vesiculosa* HM13, isolated from the intestine of a horse mackerel as a prospective host for a low-temperature secretory protein expression system, produced a single major secretory protein, P49, of unknown function in the culture supernatant. Analysis using sucrose density gradient ultracentrifugation indicated that P49 is a cargo protein carried by EMVs. *S*. *vesiculosa* HM13 displayed extensive blebbing on the surface of the outer membrane, and the size of blebs was comparable to that of EMVs. These blebs are thought to be precursors of the EMVs. Disruption of the P49 gene resulted in only a marginal decrease in the EMV production, indicating that the EMVs are produced even in the absence of the major cargo protein. Whole genome sequencing of *S*. *vesiculosa* HM13 revealed that this bacterium has a gene cluster coding for a non-canonical type II protein secretion system (T2SS) homolog in addition to a gene cluster coding for canonical T2SS. The P49 gene was located downstream of the former gene cluster. To examine the role of the putative non-canonical T2SS-like translocon, we disrupted the gene coding for a putative outer membrane channel of the translocon, named GspD2. The *gspD2* disruption lead to disappearance of P49 in the EMV fraction, whereas the production of EMVs was not significantly affected by this mutation. These results are indicative that the T2SS-like machinery functions as a novel type of protein translocon responsible for selective cargo loading to the EMVs. We also found that GFP fused to the C-terminus of P49 expressed in *S. vesiculosa* HM13 was transported to EMVs, indicating that P49 is useful as a carrier to deliver the fusion partner to EMVs. These findings deepen our understanding of the mechanism of biogenesis of EMVs and facilitate their applications.

## Introduction

Cold-adapted bacteria inhabit low-temperature environments—the deep sea, high mountains, polar regions, etc.—that account for close to 80% of the Earth’s biosphere. These bacteria have potential applications in various industrial and biotechnological fields (Feller and Gerday, 2003; Cavicchioli, 2006; Huston, 2008). Cold-active enzymes such as proteases, amylases, lipases, and cellulases produced by these bacteria have relatively high catalytic activities even at temperatures close to 0°C making them valuable tools for food and dairy engineering, wastewater treatment, and the textile industry to name a few (Gerday et al., 2000; Russell, 2000; Georlette et al., 2004; Margesin and Feller, 2010). The use of such cold-adapted bacteria as a platform for protein expression could be useful for the production of various recombinant proteins. Protein expression at low temperatures can be expected to alleviate problems associated with heat denaturation of thermolabile proteins (Feller, 2010). A low-temperature expression system is also useful for the production of enzymes that can harm the host cell, such as proteases that degrade the essential components of the cells, because activities of such enzymes can be suppressed by lowering the temperature (Georlette et al., 2004). This system would also be favorable for the overproduction of proteins that tend to form inclusion bodies, the formation of which can sometimes be avoided by lowering the cultivation temperature (Vasina and Baneyx, 1997; Duilio et al., 2004). We previously constructed a low-temperature protein expression system using an Antarctic cold-adapted bacterium, *Shewanella livingstonensis* Ac10, and found this system to be suitable for the production of thermolabile enzymes (Miyake et al., 2007). To further improve the low-temperature protein expression system, we began the search for novel cold-adapted bacteria that may be suitable as the host for secretory production of foreign proteins. Because secreted proteins can be separated from cellular proteins of the host by simple filtration or centrifugation, foreign proteins of high purity can easily be obtained. Novel strains were searched for in this study because the strain used in the previous study does not produce proteins in the extracellular milieu abundantly.

In this paper, we describe identification and characterization of a unique protein secretion system in a cold-adapted bacterium, *Shewanella vesiculosa* HM13, isolated from the intestine of a horse mackerel during a screening for the above-mentioned purpose. We found that this strain produces a single major secretory protein carried as a cargo by the extracellular membrane vesicles (EMVs). EMVs produced by Gram-negative bacteria generally have a spherical structure surrounded by lipid membranes with a size ranging from 20 to 250 nm in diameter (Kulp and Kuehn, 2010; Schwechheimer and Kuehn, 2015). They mainly contain lipopolysaccharides, phospholipids, outer membrane proteins, and periplasmic contents. The inner membrane and cytoplasmic contents including DNA and RNA have also been found in EMVs from various bacterial species (Lee et al., 2008; Pérez-Cruz et al., 2013; Sjöström et al., 2015). The molecular composition of these EMVs is remarkably different from that of the cells, with specific molecules being enriched in EMVs (Chutkan et al., 2013). This implies operation of a cargo selection mechanism for EMVs. EMVs are involved in cellular activities including intercellular communication, horizontal gene transfer, biofilm formation, infection, and defense against bacteriophages (Kulp and Kuehn, 2010; Toyofuku et al., 2015). EMVs have also been a subject of considerable interest for biotechnological applications as a platform for the secretory production of proteins, including membrane proteins, in the extracellular space (Alves et al., 2015, 2016). The development of such an application requires a good understanding of the mechanisms of biogenesis of EMVs and of how cargo molecules are selectively transported to EMVs. Information on the mechanism of transport of individual proteins into EMVs is, however, very limited. In this study, we have characterized EMVs of *S. vesiculosa* HM13 and analyzed the mechanism of transport of the major cargo protein. Our studies indicate that a type II protein secretion system (T2SS)-like machinery plays a key role in the selective cargo secretion via EMVs. These results will contribute to our understanding of the mechanism of biogenesis of EMVs and facilitate their applications.

## Materials and Methods

### Isolation of Cold-Adapted Bacteria and Characterization of Their Secretory Proteins

Intestinal contents of horse mackerel (*Trachurus japonicus*), saury (*Cololabis saira*), and squid (*Todarodes pacificus*) were suspended in Luria–Bertani (LB) medium (pH 7.0). The supernatants of the suspensions were spread onto 1% agar plates of LB medium and incubated at 4°C for several days. Colonies that appeared on the plates were picked up and grown in 5 mL liquid LB medium at 4°C for about 48 h. The cultures were centrifuged at 6,800 × *g*, 4°C for 10 min to remove the cells, and the supernatants were centrifuged at 13,000 × *g*, 4°C for 10 min to remove any debris. The supernatants were subjected to trichloroacetic acid (TCA) precipitation, and precipitated proteins were analyzed by sodium dodecyl sulfate polyacrylamide gel electrophoresis (SDS-PAGE). The strains that produced more than about 3 mg/mL secretory proteins were selected. Bacteria of interest were identified by 16S rDNA sequencing using the primers listed in Supplementary Table S1. As to the strain HM13, the average nucleotide identity analysis was performed with the ANI calculator^1^. The phylogenetic analyses were performed at TechnoSuruga Laboratory Co., Ltd. (Shizuoka, Japan).

### Cultivation of Bacterial Strains

The strains used in this study are listed in Supplementary Table S2. All the strains were grown aerobically in 5 mL liquid LB medium unless otherwise stated. When required, antibiotics were added to the medium at the following concentrations: rifampin, 50 μg/mL; kanamycin, 50 μg/mL; and chloramphenicol, 30 μg/mL. Cultivation temperature was as follows: 4 or 18°C for *S. vesiculosa* HM13 and *S. livingstonensis* Ac10, 30°C for *Shewanella oneidensis* MR-1, and 37°C for *Escherichia coli* MG1655 and *Pseudomonas putida* KT2440. A rifampin-resistant mutant of *S*. *vesiculosa* HM13 was obtained by spontaneous mutation of the *rpoB* gene, which codes for the RNA polymerase ß subunit. Sequence analysis of the gene revealed that Ser532 of the wild-type enzyme was replaced with Tyr in the mutant. We found that growth characteristics and production of P49 as a cargo of EMVs were not affected by this mutation (data not shown). The rifampin-resistant mutant was used as the parental strain for conjugation experiments.

### N-Terminal Amino Acid Sequencing of P49

Extracellular membrane vesicles were prepared by the method described in “Preparation of EMVs” from HM13 grown in LB medium at 4°C and subjected to SDS-PAGE with a 5–20% Super Sep^TM^ Ace SDS-PAGE gel (Wako Pure Chemical Industries, Osaka, Japan). After electrophoresis, the proteins were blotted onto a PVDF membrane (Merck Millipore Co., Darmstadt, Germany). The membrane was stained with Coomassie Brilliant Blue R-250, and the P49 band was cut off from the membrane and subjected to N-terminal sequencing with PPSQ-31A (Shimadzu Corporation, Kyoto, Japan).

### Preparation of EMVs

The culture (4 mL) was centrifuged at 6,800 × *g* for 10 min at 4°C to remove the cells, and the supernatant was centrifuged again at 13,000 × *g* for 10 min at 4°C. The supernatant thus obtained was passed through a 0.45 μm filter to remove cellular debris. Finally, the cell-free culture supernatant was ultracentrifuged at 100,000 × *g* for 2 h at 4°C with Optima X (Beckman Coulter, Inc., Brea, CA, United States). The pellets (EMV fraction) were suspended in 1 mL DPBSS [Dulbecco’s PBS (2.7 mM KCl, 8.9 mM Na_2_HPO_4_⋅7H_2_O, 1.5 mM KH_2_PO_4_, and 135.9 mM NaCl, pH7.2) with 0.2 M NaCl] (Chutkan et al., 2013). The suspension was used in the subsequent experiments as EMVs. The absence of the cells in this preparation was confirmed by the lack of colony formation on the LB plates at 18°C and by transmission electron microscopy (TEM) analysis. The supernatant after ultracentrifugation to remove EMVs was used as the post-vesicle fraction (PVF).

### Sucrose Density Gradient Ultracentrifugation of EMVs

EMVs were layered on a sucrose density gradient consisting of 6.25 mL each of 20, 32.5, 45, 57.5, and 70% w/v sucrose in DPBSS and subjected to ultracentrifugation at 103,745 × *g* for 15 h at 4°C (SW28 rotor, Beckman Coulter, Inc.). After ultracentrifugation, fractions (1 mL each) were collected from the bottom to the top. Proteins in each fraction were concentrated by TCA precipitation and analyzed by SDS-PAGE.

### Protease Susceptibility Test for P49

EMVs containing P49 were incubated with 1% Triton X-100 in DPBSS for 12 h for solubilization and centrifuged at 20,000 × *g* for 1 h at 4°C to remove insoluble debris. The soluble supernatant was treated with trypsin (final concentration of 0.5 μg/mL, Promega, Madison, WI, United States) at 18°C for 1, 2, 5, and 15 h. EMVs without solubilization with Triton X-100 were subjected to the same treatment. The trypsin reactions were stopped with phenylmethylsulfonyl fluoride (PMSF) at a final concentration of 10 mM, and the proteins were analyzed by SDS-PAGE.

### Quantification of EMVs

*S. vesiculosa* HM13, *S. livingstonensis* Ac10, *S. oneidensis* MR-1, *E. coli* MG1655, and *P. putida* KT2440 were harvested at the stationary phase (OD_600_ = 3.0), and their EMVs were collected as described above. EMVs were quantified as described (McBroom et al., 2006) by staining with a lipophilic dye, FM4–64 (Molecular Probes/ThermoFisher, Chicago, IL, United States), at a final concentration of 5 μg/mL in DPBSS for 20 min at room temperature. As a negative control, DPBSS was used. The fluorescence intensity of FM4-64 in the membrane was measured with excitation at 515 nm and emission at 635 nm with an RF5300-PC spectrofluorophotometer (Shimadzu Corporation).

### Phospholipid and Fatty Acyl Chain Analysis by ESI-MS and GC-MS

Phospholipids were extracted from EMVs and the cells with methanol/chloroform (2:1, vol/vol) by the Bligh and Dyer procedure (Bligh and Dyer, 1959). The extracts were analyzed by ESI-MS with a triple-quadrupole Sciex API 3000 LC/MS/MS System (Applied Bio-Systems, Foster City, CA, United States) equipped with an ionspray ion source in the negative mode (Cho et al., 2012). The ionspray voltage was −4,200 kV. The spectra were recorded in the range of *m*/*z* 600–800. For fatty acyl chain analysis, the phospholipid extracts were methyl-esterified as described previously (Tokunaga et al., 2017). The methyl-esterified samples were analyzed with a Clarus 680 gas chromatograph coupled with a Clarus SQ 8C mass spectrometer (Perkin-Elmer, Wellesley, MA, United States) equipped with an Agilent J&W GC column DB-1 (Agilent Technologies, Inc., Santa Clara, CA, United States).

### Targeted Gene Disruption in *S. vesiculosa* HM13

Genes coding for P49 and GspD2 (a putative outer membrane conduit for P49) of *S. vesiculosa* HM13 were disrupted individually by integration of a gene knockout plasmid, pKNOCK-Km^r^, as previously reported (Cho et al., 2012). About 500-bp internal region of the respective genes and linearized pKNOCK-Km^r^ were amplified by PCR using the primers listed in Supplementary Table S1. The PCR products were assembled by using NEBuilder HiFi DNA Assembly Master Mix (New England BioLabs, Japan, Inc., Tokyo, Japan) and introduced into competent *E. coli* S17-1/λ*pir* cells. Then, the plasmid was transferred to the rifampin-resistant mutant of *S. vesiculosa* HM13 (HM13-Rif^r^) by conjugation at 18°C on LB plates. Single crossover recombinants were selected on LB plates containing kanamycin (50 μg/mL) and rifampin (50 μg/mL). Representative colonies of each mutant were analyzed by PCR to verify the gene disruption.

For complementation of the *gspD2* disruption, an expression plasmid carrying *gspD2* (pJRD215-Cm^r^-p*gspD2*) was constructed as follows. Linearized pJRD215-Cm^r^, the DNA fragment containing the *gspD2*-coding region, and the upstream flanking region (1 kbp) of the P49 gene containing its predicted promoter were amplified by using the primer set of pJRD215-fwd and pJRD215-rev, the set of gspD2-fwd and gspD2-rev, and the set of up1000-fwd and up1000-rev, respectively. The PCR products were assembled using NEBuilder HiFi DNA Assembly Master Mix to obtain pJRD215-Cm^r^-p*gspD2* and introduced into *E. coli* S17-1/λ*pir* cells. The plasmid was transferred from the recombinant *E. coli* S17-1/λ*pir* cells to the *gspD2*-disrupted mutant, Δ*gspD2*, of *S. vesiculosa* HM13 by conjugation at 18°C on LB plates, and Δ*gspD2* harboring pJRD215-Cm^r^-p*gspD2* was selected by using LB plates containing kanamycin (50 μg/mL), rifampin (50 μg/mL), and chloramphenicol (30 μg/mL). Introduction of the plasmid into Δ*gspD2* was confirmed by colony PCR by using the primer set of pJRD215 mluI-fwd and pJRD215 speI-rev.

### Construction of Recombinant *S. vesiculosa* HM13 Strains That Produce GFP and P49-Fused GFP

The recombinant *S. vesiculosa* HM13 strain (P49-GFP) that produces GFP fused to the C-terminus of P49 was constructed by integrating the GFP gene at the 3′-terminus of the P49 gene in the genome as follows. About 500-bp DNA fragment coding for the C-terminal region of P49 without stop codon, the GFP gene, and linearized pKNOCK-Km^r^ were amplified by PCR using the primers listed in Supplementary Table S1. As a template for the GFP gene amplification, pGreen was used (Supplementary Table S2). The PCR products were assembled by using NEBuilder HiFi DNA Assembly Master Mix and introduced into competent *E. coli* S17-1/λ*pir* cells. Then, the plasmid was transferred to HM13-Rif^r^ by conjugation at 18°C on LB plates. Single crossover recombinants were selected on LB plates containing kanamycin (50 μg/mL) and rifampin (50 μg/mL). Representative colonies were analyzed by PCR to verify insertion of the GFP gene at the 3′-terminus of the P49 gene by using the primer set of P49C-fwd and pKNOCK check-rev.

The recombinant *S. vesiculosa* HM13 strain (ΔP49/pGFP) that produces GFP without being fused to P49 was constructed as follows. Linearized pJRD215-Cm^r^, the GFP gene, and the upstream flanking region (500 bp) of the P49 gene containing its predicted promoter were amplified by using the primers listed in Supplementary Table S1. The PCR products were assembled using NEBuilder HiFi DNA Assembly Master Mix to obtain pJRD215-Cm^r^-pGFP. The plasmid was introduced into *E. coli* S17-1/λ*pir* cells and transferred to ΔP49 by conjugation at 18°C on LB plates. The recombinant ΔP49 harboring pJRD215-Cm^r^-pGFP (ΔP49/pGFP) was selected by using LB plates containing kanamycin (50 μg/mL), rifampin (50 μg/mL), and chloramphenicol (30 μg/mL). Introduction of the plasmid was verified by colony PCR by using the primer set of pJRD215 mluI-fwd and pJRD215 speI-rev.

### TEM Analysis

EMVs were visualized by TEM as previously reported (Yokoyama et al., 2017). Two microliters of the EMV samples were adsorbed onto hydrophilized carbon-coated copper grids and then treated twice with 2% uranyl acetate to negatively stain the samples. The TEM images were obtained with a JEM-1400 transmission electron microscope (JEOL, Ltd., Tokyo, Japan) at an accelerating voltage of 120 kV. Images were acquired using a charge-coupled device (CCD) camera (a built-in camera in the JEM-1400).

The intact structure of EMVs in buffer was visualized by cryo-electron microscopy (cryo-EM) at −175°C with a side-entry type cryo-specimen holder (Model 626.DH holder, Gatan Inc., Pleasanton, CA, United States). The samples were loaded onto microgrids (lacey carbon film, Ted Pella Inc., Redding, CA, United States) and treated using the same procedures as described previously (Yokoyama et al., 2017).

### Field Emission-Scanning Electron Microscopic Analysis

To observe the surface morphology of the cells secreting EMVs, *S. vesiculosa* HM13 (wild type) was statically grown in 1 mL liquid LB medium at 18°C until the log phase (OD_600_ = 1.0). To confirm the intactness of the cells obtained under this condition, the cell viability test was performed by using LIVE/DEAD BacLight Bacterial Viability Kit (Molecular Probes/ThermoFisher), which showed that 97.2 ± 0.1% of the cells were viable. The culture was fixed with glutaraldehyde solution at a final concentration of 1%. Field emission-scanning electron microscopy (FE-SEM) analysis was performed according to the method (Koyama et al., 2015) with a slight modification. Samples were coated with osmium using Osmium Plasma Coater (POC-3, Meiwa Shoji Co., Osaka, Japan) and observed with a field-emission scanning electron microscope, JSM-6700F (JEOL, Tokyo, Japan) at an acceleration voltage of 5 kV.

### Fractionation of the Cells and Culture Supernatant and Western Blot Analysis

*S. vesiculosa* HM13-Rif^r^, ΔP49, Δ*gspD2*, Δ*gspD2* harboring pJRD215-Cm^r^-p*gspD2*, ΔP49/pGFP (ΔP49 harboring pJRD215-Cm^r^-pGFP), and P49-GFP (recombinant HM13-Rif^r^ that produces GFP fused to the C-terminus of P49) were grown at 18°C in LB medium containing appropriate antibiotics to an OD_600_ of 2.5. The culture was centrifuged at 6,800 × *g* for 10 min at 4°C to pellet the cells and centrifuged again at 13,000 × *g* for 10 min at 4°C to collect the supernatant. The pelleted cells were suspended in DPBSS and disrupted by sonication. Low-speed centrifugation (6,800 × *g*) was performed at 4°C for 10 min to remove undisrupted cells. Soluble protein fraction and insoluble protein fraction of the cells were separated by ultracentrifugation at 100,000 × *g* for 2 h at 4°C with an Optima X (Beckman Coulter, Inc.). Culture supernatant was ultracentrifuged at 100,000 × *g* for 2 h at 4°C with an Optima X (Beckman Coulter, Inc.) to pellet EMVs and obtain PVF as the supernatant. The EMVs and the insoluble protein pellet were suspended in DPBSS. All the samples were mixed with 1/10 volume of 100% (w/v) TCA, incubated on ice for 15 min, and subsequently centrifuged at 20,500 × *g* for 15 min at 4°C for the precipitation of proteins. The pellets were washed twice with ice-cold acetone, air-dried for 5 min at room temperature, and suspended in 12 mM Tris-HCl (pH 6.8) buffer containing 10% glycerol, 0.4% sodium dodecyl sulfate, 0.02% bromophenol blue, and 2.88 mM 2-mercaptoethanol.

The protein samples corresponding to 100 μL culture were separated on a 5–20% SuperSep^TM^ Ace SDS-PAGE gel (Wako Pure Chemical Industries) and blotted onto a PVDF membrane (Merck Millipore Co.). P49 was detected by using polyclonal anti-P49 rabbit antiserum raised against the peptide ASDRGFVSNTSNGS corresponding to the sequence in the close vicinity of the C-terminus of P49. The antiserum was purchased from Eurofins Genomics (Tokyo, Japan). The antiserum was diluted to 1:50,000. Omp74 of *S. vesiculosa* HM13, sharing 89.7% amino acid sequence identity with Omp74 of *S. livingstonensis* Ac10, was detected with previously prepared polyclonal rabbit antiserum raised against Omp74 of *S. livingstonensis* Ac10 (Dai et al., 2012). The antiserum was diluted to 1:40,000. GFP was detected with 10,000-fold diluted anti-GFP antibody purchased from GeneTex (Irvine, CA, United States). Blotting Grade Affinity Purified Goat Anti-Rabbit IgG (H + L)-HRP Conjugate (Bio-Rad Laboratories, Inc., Hercules, CA, United States) was used as a secondary antibody at a final dilution of 1:50,000. The immunoblot detection was performed using the Chemi-Lumi One Ultra (Nacalai Tesque, Kyoto, Japan) and a C-Digit Blot Scanner (LI-COR Biosciences, Lincoln, NE, United States).

### Iodixanol Density Gradient Centrifugation

The EMV fraction of *S. vesiculosa* HM13-Rif^r^ and PVF of Δ*gspD2* were subjected to ultracentrifugation (100,000 × *g* for 12 h at 4°C; SW50.1 rotor, Beckman Coulter, Inc.) in a discontinuous iodixanol (OptiPrep^TM^, Axis-Shield Poc AS, Oslo, Norway) density gradient with 0.8 mL of 60%, 0.8 mL of 50%, 0.8 mL of 40%, 0.8 mL of 30%, and 0.8 mL of 20% iodixanol. Applied on top of the gradient was the EMV fraction of HM13-Rif^r^ prepared from a 10 mL culture and concentrated to 0.3 mL or PVF of Δ*gspD2* prepared from a 4 mL culture and concentrated to 0.3 mL with Amicon Ultra filters (Merck Millipore Co.; 10 kDa molecular weight cut-off). After ultracentrifugation, fractions (0.4 mL each) were collected from the top and subjected to SDS–PAGE and western blot analysis.

## Results

### Isolation and Identification of *S. vesiculosa* HM13

We screened cold-adapted microorganisms from fish intestine that could potentially be useful as a host for secretory protein production at low temperatures as described in Section “Materials and Methods.” As the result, we obtained several bacterial strains that belonged to the genera *Pseudomonas*, *Shewanella*, and *Flavobacterium* (data not shown). Specifically, a bacterial strain that was isolated from the intestine of a horse mackerel and that displayed unusual protein secretion characteristics (occurrence of a single major protein) as described below was further studied.

**FIGURE 1:**
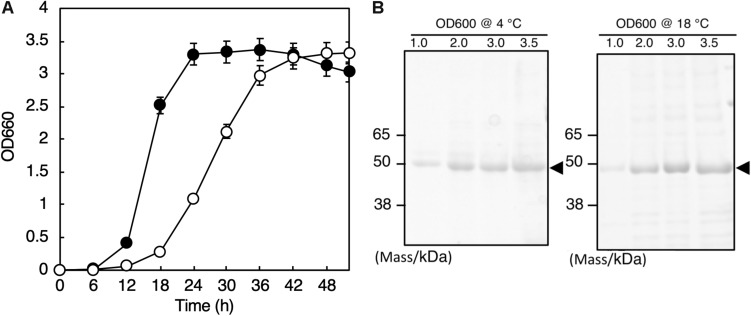
Growth and secretory protein production of *S. vesiculosa* HM13. **(A)** The cells were grown at 4°C (open circles) and 18°C (closed circles) in LB medium. Bars represent the SD values calculated from three independent experiments. **(B)** The cells were gown at 4 and 18°C to the indicated OD_600_, and 1 mL aliquots of the culture supernatants were subjected to TCA precipitation and SDS-PAGE. The gel was stained with Coomassie Brilliant Blue G-250. The major protein bands of about 49 kDa are indicated by arrowheads.

The whole genome sequence of this isolate was determined by the method described in Hirose et al. (2016), and we found the 16S rRNA genes (Accession numbers: LC460999 and LC461000) in the genome. Phylogenetic analysis with the 16S rRNA gene sequences and the following average nucleotide identity analysis with the whole genome sequence revealed that the isolate belongs to *Shewanella vesiculosa*. Thus, we named this isolate *S. vesiculosa* HM13 (Horse Mackerel strain no. 13). The strain was deposited in Japan Collection of Microorganisms (JCM), RIKEN BioResource Research Center, and the strain number “JCM 33296” was provided. *S. vesiculosa* HM13 was found to grow in the temperature range of 4–25°C, and the optimum growth was observed at 18°C (data not shown). The doubling time was 4.1 h at 4°C and 1.8 h at 18°C (Figure 1A).

### Secretory Protein Production by *S. vesiculosa* HM13

We found that *S. vesiculosa* HM13 secreted a single major protein with an apparent molecular mass of about 49 kDa at both 4 and 18°C (Figure 1B). We named this protein P49. The amounts of P49 as well as other minor proteins in the culture supernatant were found to increase as the cells grew. The yields of P49 in late-stationary phase cultures (OD_600_ = 3.0) determined by quantification of its band intensity were 3.6 mg/L at 4°C and 5.3 mg/L at 18°C. Considering its high purity in the culture supernatant, P49 could be useful as a carrier for secretory protein production using this strain as a host at low temperatures: a foreign protein fused with P49 is expected to be delivered to the extracellular milieu.

The N-terminal amino acid sequence of P49 was determined to be GTFTGVKNAAGTVVSNENVLIY. In 4,877,090 bp of the total genome of this strain, 4,279 coding sequences were annotated (Rapid Annotation using Subsystem Technology ver. 2.0)^2^. A search for the gene encoding the N-terminal amino acid sequence of P49 resulted in the identification of HM3347 as the P49-encoding gene (DDBJ: LC431027). The N-terminal 22 amino acid residues of the predicted precursor of P49 were thought to be cleaved off during maturation resulting in the predicted mature form of P49 composed of 451 amino acid residues. A BLAST search^3^ revealed Ssed_3019 (GenBank: ABV37623), a hypothetical protein of *Shewanella sediminis* HAW-EB3, to display the highest degree of similarity to P49. The amino acid sequence identity between these two proteins was 28%. No other proteins were found to display significant similarity to P49 along the entire length of the protein. P49 is, therefore, a novel protein the function of which cannot be predicted based on its sequence.

### Production of EMVs Harboring P49 by *S. vesiculosa* HM13

Prediction of membrane-spanning regions using the TMPred program revealed two putative transmembrane segments in the mature form of P49 spanning amino acid residues 250–270 and 330–354 (the numbers are indicative of amino acid positions in the precursor protein)^4^ (Hofmann and Stoffel, 1993). Analysis with the SOSUIGramN program^5^ (Imai et al., 2008) suggested its localization in the outer membrane. Indeed, fractionation of the cellular membrane fraction by sucrose density gradient ultracentrifugation demonstrated co-localization of P49, an outer membrane porin homolog (Omp74), and the cell surface lipopolysaccharides (Supplementary Figures S1A,B), indicating that P49 exists in the outer membrane in addition to the culture supernatant. Thus, we speculated that P49 might be associated with membranes in the culture supernatant, most likely as a cargo of EMVs.

**FIGURE 2:**
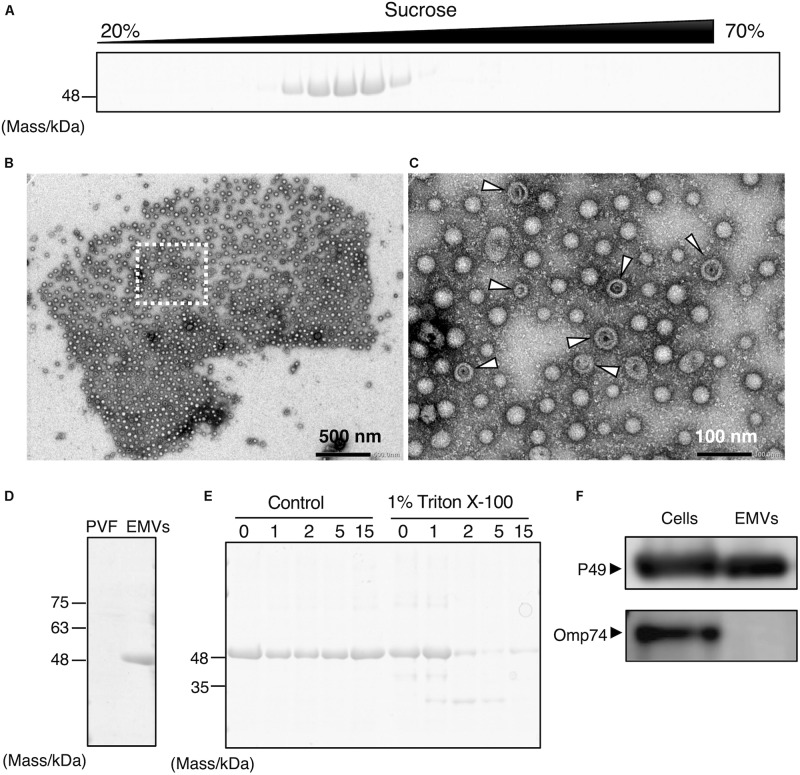
Production of P49-containing EMVs by *S. vesiculosa* HM13. **(A)** Sucrose density gradient ultracentrifugation of the culture supernatant of *S. vesiculosa* HM13 grown in LB medium at 4°C. Each fraction was subjected to SDS-PAGE. **(B)** Negative-stained TEM image of the fraction containing P49 showing the occurrence of EMVs. **(C)** Magnified view of the boxed area in **(B)**. Arrowheads indicate vesicles with abnormal morphology, which may be double-membrane-bounded vesicles that naturally occur or collapsed vesicles artificially produced by dehydration. **(D)** SDS-PAGE of EMVs prepared by ultracentrifugation of the culture supernatant of *S. vesiculosa* HM13 grown at 4°C. PVF (post-vesicle fraction) refers to the supernatant that remains after ultracentrifugation of the culture supernatant to remove EMVs as pellets. **(E)** SDS-PAGE analysis of EMVs after trypsin treatment. EMVs prepared by ultracentrifugation were treated with 0.5 μg/mL trypsin in the presence or absence of Triton X-100 for the indicated times (h). **(F)** Western blot analysis of P49 and Omp74 in the cells and EMVs with an anti-P49 polyclonal antibody and an anti-Omp74 antibody, respectively. The cells were grown in LB medium at 18°C to the stationary phase. The cells and EMVs prepared from 100 μL culture were subjected to the analysis.

To determine the localization of P49, the culture supernatant of *S. vesiculosa* HM13 was subjected to sucrose density gradient ultracentrifugation. SDS-PAGE analysis of the fractions showed that P49 was recovered in the middle fractions containing 30–40% sucrose (Figure 2A). We analyzed the P49-containing fractions by TEM and found a large number of EMVs in these fractions (Figures 2B,C). Co-fractionation of P49 with EMVs suggested its association with EMVs. The diameter of EMVs from this strain was determined to be in the range of 30–100 nm. The large majority of EMVs were single-membrane-bounded (Figure 2C) although morphological variants of EMVs were also observed (Figure 2C, arrowheads).

Extracellular membrane vesicles and P49 could also be recovered as pellets by ultracentrifugation without the use of sucrose density gradients (Figure 2D). To conclusively determine if P49 is associated with EMVs, the pellets containing P49 were subjected to trypsin treatment in the presence or absence of Triton X-100 (Figure 2E). In the absence of Triton X-100, P49 was not digested, whereas digestion in the presence of 1% Triton X-100 led to almost complete disappearance of the P49 band by 2 h. This was indicative that P49 is associated with EMVs and is probably protected from protease digestion due to its being embedded in the membrane of EMVs.

We further examined whether P49 is selectively transported to EMVs or other outer membrane proteins are also transported to EMVs indiscriminately. For this purpose, we analyzed the distribution of Omp74, a homolog of a major outer membrane protein OmpA of *E. coli* (Wang, 2002). As the result, we found that Omp74 was not transported to EMVs, whereas P49 was found in EMVs (Figure 2F). Thus, we concluded that P49 is selectively transported to EMVs.

**FIGURE 3:**
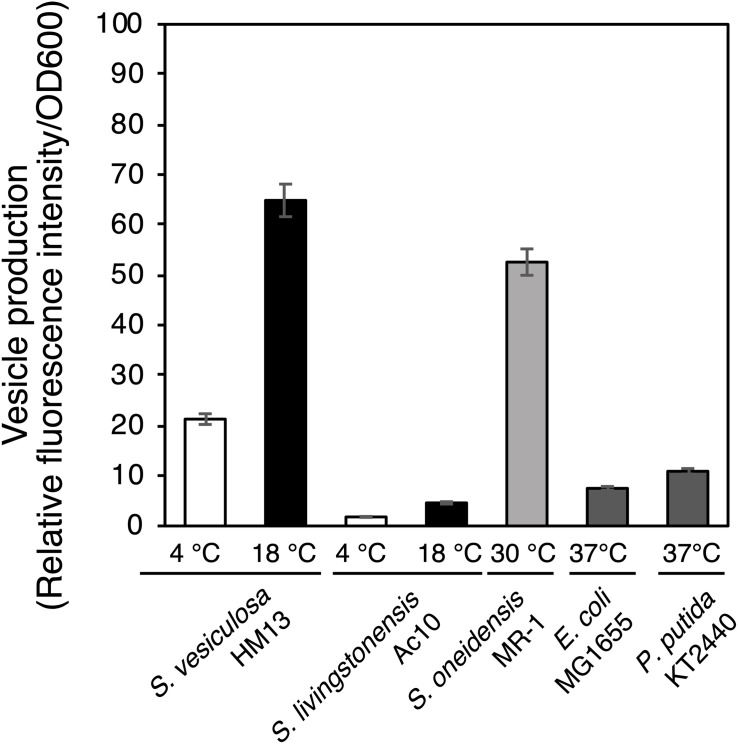
EMV production by *Shewanella* species and other Gram-negative bacteria. The cells were grown in LB medium at 4°C (white bar) or 18°C (black bar) for *S. vesiculosa* HM13 and *S. livingstonensis* Ac10, at 30°C (gray bar) for *S. oneidensis* MR-1, and at 37°C (dark gray bar) for *E. coli* MG1655 and *P. putida* KT2440. EMVs were obtained when the OD_600_ reached 3.0. *n* = 3.

We next compared EMV productivity of *S. vesiculosa* HM13 with that of other *Shewanella* species and other well-known EMV-producing Gram-negative bacteria using a lipophilic dye, FM4-64 (Figure 3). *S. vesiculosa* HM13 and a related cold-adapted strain, *S. livingstonensis* Ac10, were grown at 4 and 18°C. The other bacteria used in this analysis were cultured at their respective optimal temperatures for growth. EMV production by *S. vesiculosa* HM13 at 18°C was found to be nearly three times greater than that observed at 4°C. At both 4°C and 18°C, *S. vesiculosa* HM13 produced much larger amounts of EMVs than *S. livingstonensis* Ac10 as noted from the fluorescence intensity of FM4-64. In addition, production of EMVs by *S. vesiculosa* HM13 at 18°C was higher than that by *S. oneidensis* MR-1, *P. putida* KT2440, and *E. coli* MG1655.

### Characterization of EMVs of *S. vesiculosa* HM13

**FIGURE 4:**
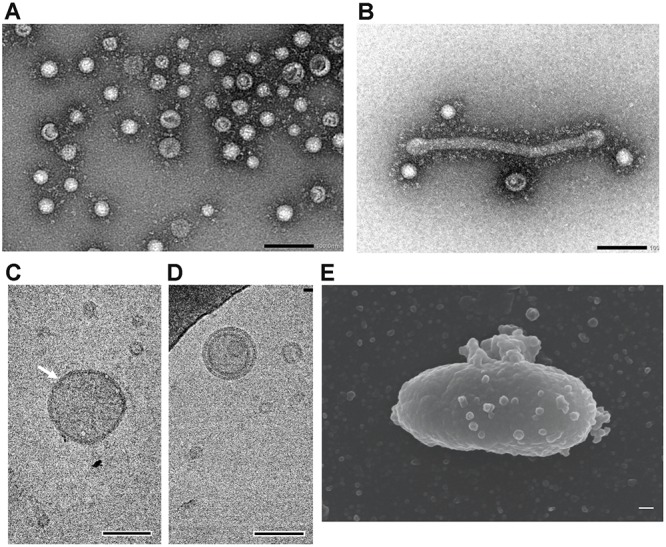
Morphological characterization of EMVs from *S. vesiculosa* HM13. **(A)** Negative-stained TEM image of EMVs from *S. vesiculosa* HM13 grown at 4°C. **(B)** Negative-stained TEM image of tube-like structure observed in the EMV fraction of *S. vesiculosa* HM13 grown at 4°C. **(C,D)** Cryo-EM images of EMVs from *S. vesiculosa* HM13 grown at 4°C. An arrow indicates EMV surrounded by a high electron density region. **(E)** FE-SEM image of the *S. vesiculosa* HM13 cell grown at 18°C showing many buds appearing on the cell surface. Bars represent 100 nm.

Isolated EMVs were negatively stained and examined by TEM. The majority of EMVs of *S. vesiculosa* HM13 were spherical; tube-like structures were also observed, although much less frequently, in the same fraction (Figures 4A,B). The diameter of the spherical EMVs as observed by TEM varied from 30 to 100 nm, whereas the average diameter measured by dynamic light scattering (DLS) analysis was between 80 and 100 nm (Supplementary Figure S2). Analysis by cryo-EM showed a relatively high electron density region surrounding EMVs, which is supposed to represent the bilayer membrane structure (Figure 4C). EMVs with double-membrane-bounded structure were also observed (Figure 4D). Examination of *S. vesiculosa* HM13 by FE-SEM revealed many buds on the cell surface; the size of these buds was comparable to that of the EMVs, suggesting that EMVs are secreted from the cell surface without cell lysis (Figure 4E). DLS analysis and TEM observation demonstrated that morphologies of the majority of EMVs from cells grown at 4°C and 18°C were not significantly different from each other (Supplementary Figures S2, S3A–C and Figures 4A,C,D).

**FIGURE 5:**
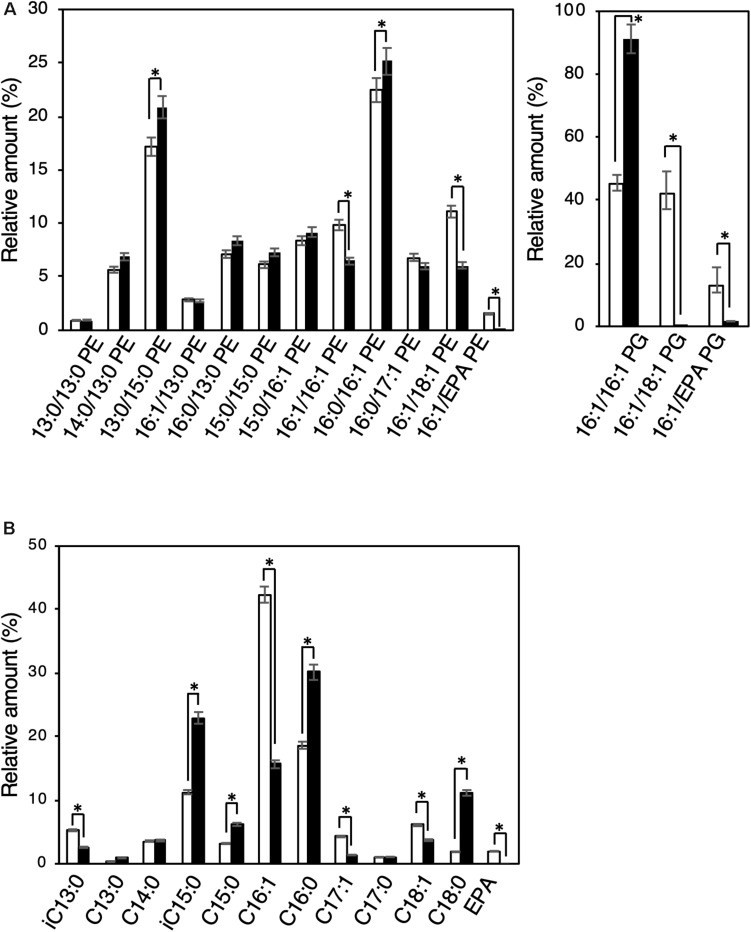
Phospholipid and fatty acyl chain compositions of EMVs and the cells of *S. vesiculosa* HM13. **(A)** The cells were grown in LB medium at 4°C to the stationary phase. EMVs were obtained from the culture supernatant by ultracentrifugation. Phospholipids were extracted from the cells (white bar) and EMVs (black bar) by the Bligh and Dyer method (Bligh and Dyer, 1959) and subjected to electrospray ionization-mass spectrometry (ESI-MS) analysis. *n* = 3, ^∗^*p* < 0.001 (Student’s *t*-test). **(B)** Methyl-esterified fatty acids were prepared from the phospholipid extracts and analyzed by GC-MS. *n* = 3, ^∗^*p* < 0.001 (Student’s *t*-test). EPA indicates eicosapentaenoic acid.

Biochemical characterization of EMVs was performed by analyzing and comparing the constituent phospholipids [phosphatidylethanolamine (PE) and phosphatidylglycerol (PG)] and their fatty acyl chains between EMVs and the cells of *S. vesiculosa* HM13 (Figure 5). The major phospholipid species in the cells and EMVs were 16:0/16:1 PE and 13:0/15:0 PE for PEs and 16:1/16:1 PG for PGs (Figure 5A). Relative amounts of phospholipids containing unsaturated fatty acyl chains both at *sn*-1 and *sn-*2 positions of the glycerol backbone—16:1/16:1 PE, 16:1/18:1 PE, 16:1/eicosapentaenoic acid (EPA) PE, 16:1/18:1 PG, and 16:1/EPA PG—were significantly lower in the EMVs than in the cells (*p* < 0.001, Student’s *t*-test).

Gas chromatography-mass spectrometry (GC-MS) analysis was performed for the methyl-esterified fatty acids prepared from the phospholipid extracts of *S. vesiculosa* HM13 cells as well as EMVs. Major fatty acyl groups in the phospholipid extracts of cells were the palmitoleoyl (C16:1), palmitoyl (C16:0), and iso-pentadecanoyl (iC15:0) groups accounting for 42.3, 18.7, and 11.3%, respectively, of the total fatty acyl groups (Figure 5B). Similar analysis with the EMVs revealed that major fatty acyl groups were the palmitoyl (C16:0), iso-pentadacanoyl (iC15:0), palmitoleoyl (C16:1), and stearoyl (C18:0) groups accounting for 30.1, 23.0, 15.7, and 11.2%, respectively, of the total fatty acyl groups. Compared to the cells, the EMVs were found to contain relatively large amounts of saturated fatty acyl groups, particularly the palmitoyl (C16:0), iso-pentadecanoyl (iC15:0), and stearoyl (C18:0) groups.

### Organization and Characteristics of the Gene Cluster Containing the P49 Gene

**FIGURE 6:**
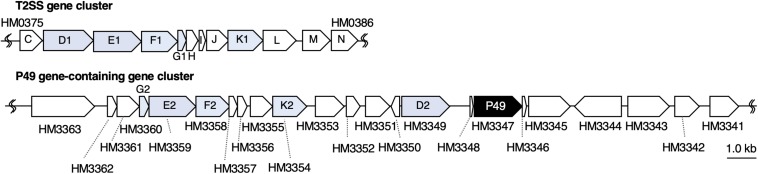
Organization of the gene cluster containing the P49 gene. Maps of the gene cluster containing the P49 gene and that coding for the canonical T2SS of *S. vesiculosa* HM13. Characteristics of the genes constituting these clusters are summarized in Tables 1, 2, respectively. The gene coding for P49, HM3347, is located downstream of the genes coding for homologs of the components of bacterial T2SS. These homologs were named GspG2 (HM3360), GspE2 (HM3359), GspF2 (HM3358), GspK2 (HM3354), and GspD2 (HM3349), whereas their counterparts constituting the canonical T2SS were named GspG1 (HM0379), GspE1 (HM0377), GspF1 (HM0378), GspK1 (HM0383), and GspD1 (HM0376), respectively.

To understand the molecular mechanism of P49 loading onto EMVs, we analyzed the gene cluster containing the P49 gene to determine if the neighboring genes are involved in this process. Analysis of the whole genome sequence of *S. vesiculosa* HM13 revealed that the gene coding for P49, designated HM3347, is located within a gene cluster containing genes that encode homologs of the T2SS components (HM3349, HM3354, HM3358, HM3359, and HM3360) (Figure 6 and Table 1). T2SS is a secretion system used by Gram-negative bacteria for the translocation of proteins to the extracellular milieu (Thomassin et al., 2017). Further analysis of the genome of this bacterium has revealed components of a canonical T2SS of the general secretory pathway encoded by a different gene cluster consisting of the genes designated HM0375–HM0386 (Figure 6 and Table 2). The T2SS machinery of Gram-negative bacteria typically comprises trans-envelope connections (GspCLM), an outer membrane channel (GspD), a secretion ATPase (GspE), an inner membrane platform protein (GspF), pseudopilins (GspGK), minor pseudopilins (GspHIJ), and an inner membrane protein of unknown function (GspN) (Thomassin et al., 2017). Genes coding for seven of these components (GspCLM, GspHIJ, and GspN) are missing in the P49 gene-containing cluster, whereas the genes coding for these components were found in the other gene cluster. Therefore, the P49 gene-containing cluster probably codes for proteins that constitute a T2SS-like protein translocon that is distinct from the canonical T2SS. The gene cluster coding for a similar T2SS-like translocon, which lacks the genes coding for GspCLM, GspHIJ, and GspN, was found from at least eight strains, including two *Shewanella* strains, *Shewanella* sp. UCD-KL12 (GeneBank ID: MPHJ00000000, Lujan et al., 2017) and *Shewanella waksmanii* ATCC BAA-643 (GenBank assembly accession: GCA_000518805), two *Colwellia* strains (GenBank assembly accession: GCA_002104455.1 and NCBI Reference Sequence: NZ_NBOF01000001.1), and three *Pseudoalteromonas* strains (GeneBank ID: JWIH00000000, GenBank ID: CP021646 and CP021647, and DDBJ accession number: PRJDB4170). It should be examined in future studies whether the T2SS-like protein translocon contains GspCLM, GspHIJ, and GspN encoded by the canonical T2SS gene cluster or their counterparts or it functions without involvement of these components. The proteins encoded by the P49 gene-containing cluster that share homology with canonical T2SS components were designated GspG2 (HM3360), GspE2 (HM3359), GspF2 (HM3358), GspK2 (HM3354), and GspD2 (HM3349), whereas their homologs constituting the canonical T2SS were named GspG1 (HM0379), GspE1 (HM0377), GspF1 (HM0378), GspK1 (HM0383), and GspD1 (HM0376), respectively. The amino acid sequence identities between these homologs were 31.9, 34.9, 26.6, 16.2, and 15.6%, respectively. The function of the putative T2SS-like protein translocon was examined in the following experiments.

**TABLE 1:** Predicted functions and localization of proteins encoded by the gene cluster containing the P49 gene of *S. vesiculosa* HM13.

**Gene**	**Function of the protein predicted by its sequence**	Localization*	**Accession**
HM3363	Wza, EpsE, polysaccharide export protein	OM	LC431043
HM3362	WecA, enzyme of enterobacterial common antigen biosynthesis, undecaprenyl-phosphate alpha-*N*-acetylglucosaminyl 1-phosphatetransferase	IM	LC431042
HM3361	WecA, enzyme of enterobacterial common antigen biosynthesis, undecaprenyl-phosphate alpha-*N*-acetylglucosaminyl 1-phosphatetransferase	IM	LC431041
HM3360	GspG2, Type II secretion system, major pseudopilin	IM	LC431040
HM3359	GspE2, Type II secretion system, secretion ATPase	CP	LC431039
HM3358	GspF2, Type II secretion system, inner membrane platform protein	IM	LC431038
HM3357	Prepilin-type cleavage/methylation domain-containing protein	ND	LC431037
HM3356	Prepilin-type cleavage/methylation domain-containing protein	ND	LC431036
HM3355	Prepilin-type cleavage/methylation domain-containing protein	ND	LC431035
HM3354	GspK2, Type II secretion system, minor pseudopilin	IM	LC431034
HM3353	Hypothetical protein, no putative conserved domains	ND	LC431033
HM3352	Hypothetical protein, no putative conserved domains	IM	LC431032
HM3351	Hypothetical protein, no putative conserved domains	ND	LC431031
HM3350	Hypothetical protein, no significant similarity found	ND	LC431030
HM3349	GspD2, Type II secretion outer membrane pore forming protein, secretin	OM	LC431029
HM3348	Hypothetical protein, no significant similarity found	ND	LC431028
HM3347	P49, no putative conserved domains, a cargo protein of EMVs	ND	LC431027
HM3346	Hypothetical protein, no putative conserved domains	ND	LC431026
HM3345	GDPD, uncharacterized hypothetical proteins similar to the catalytic domains of phosphoinositide-specific phospholipase and glycerophosphodiester phosphodiesterases	IM	LC431025
HM3344	OpgE, phosphoethanolamine transferase for periplasmic glucans (OPG), alkaline phosphatase superfamily	IM	LC431024
HM3343	Wzx, a subfamily of the multidrug and toxic compound extrusion (MATE)-like proteins, flippase assisting in the membrane translocation of lipopolysaccharides including those containing O-antigens	IM	LC431023
HM3342	NfnB, nitroreductase family protein	CP	LC431022
HM3341	Ugd, UDP-glucose 6-dehydrogenase	CP	LC431021

*^∗^Protein localization was predicted with PSORTb version 3.0.2 (http://www.psort.org/psortb/). OM, outer membrane; IM, inner membrane; CP, cytoplasm; ND, not determined.*

**TABLE 2:** Predicted functions and localization of proteins encoded by the T2SS gene cluster of *S. vesiculosa* HM13.

**Gene**	**Function of the protein predicted by its sequence**	Localization*	**Accession**
HM0375	GspC, inner membrane-anchored trans-envelope connection protein, interaction with secretin	IM	LC431044
HM0376	GspD, outer membrane conduit, secretin	OM	LC431045
HM0377	GspE, secretion ATPase	CP	LC431046
HM0378	GspF, inner membrane platform protein	IM	LC431047
HM0379	GspG, major pseudopilin	IM	LC431048
HM0380	GspH, minor pseudopilin	IM	LC431049
HM0381	GspI, minor pseudopilin	IM	LC431050
HM0382	GspJ, minor pseudopilin	IM	LC431051
HM0383	GspK, minor pseudopilin	IM	LC431052
HM0384	GspL, inner membrane-anchored trans-envelope connection protein	ND	LC431053
HM0385	GspM, inner membrane-anchored trans-envelope connection protein	ND	LC431054
HM0386	GspN, unknown function	IM	LC431055

*^∗^Protein localization was predicted with PSORTb version 3.0.2 (http://www.psort.org/psortb/). OM, outer membrane; IM, inner membrane; CP, cytoplasm; ND, not determined.*

### Involvement of the Putative T2SS-Like Translocon in the Transport of P49 to EMVs

**FIGURE 7:**
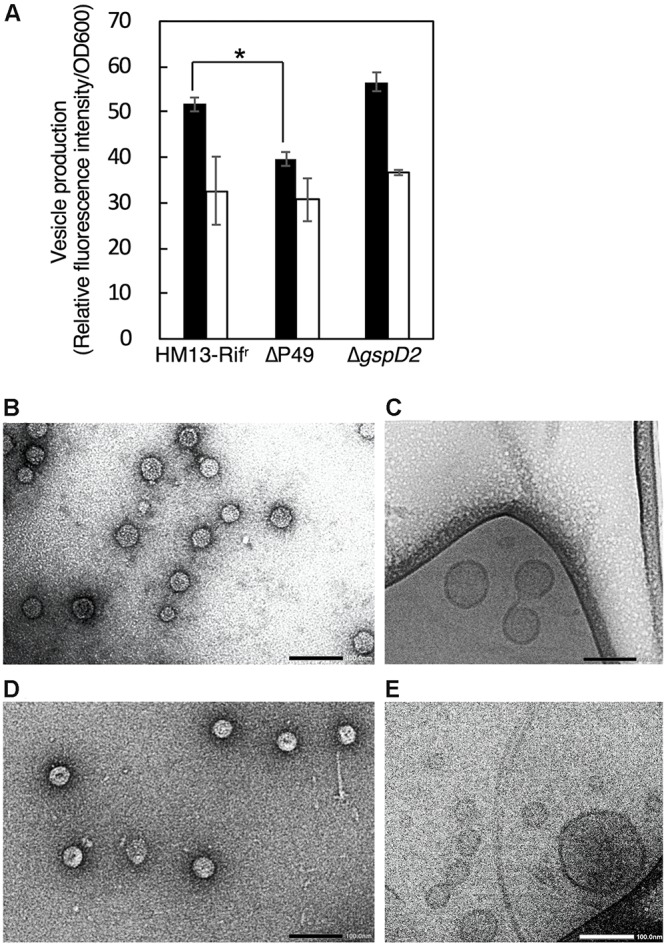
EMV production by *S. vesiculosa* HM13 mutants that lack P49 or GspD2. **(A)** Production of EMVs by *S. vesiculosa* HM13 mutants that lack either P49 or GspD2. EMVs produced by HM13-Rif^r^, the P49-lacking mutant (ΔP49), and the GspD2-lacking mutant (Δ*gspD2*) at 18°C (black) and 4°C (white) were quantified with a lipophilic dye, FM4-64. *n* = 3, ^∗^*p* < 0.001 (Student’s *t*-test). EMVs produced by ΔP49 and Δ*gspD2* at 4°C were analyzed by negative-staining TEM (**B,D**, respectively) and cryo-EM (**C,E**, respectively). Bars indicate 100 nm.

In order to investigate the physiological functions of P49 and the putative T2SS-like translocon in the production of EMVs, we disrupted the genes coding for P49 and GspD2, a homolog of the outer membrane channel of T2SS, by using the rifampin-resistant mutant of *S. vesiculosa* HM13 (HM13-Rif^r^) as the parent strain. This strain was used to facilitate isolation of the *S. vesiculosa* HM13 mutants after conjugation with *E. coli* S17-1/λ*pir*, which is sensitive to rifampin. We found that the disruption of the P49 and GspD2 genes did not affect the growth of *S. vesiculosa* HM13 (data not shown). Also, these genes were not essential for the production of EMVs, although disruption of the P49 gene caused a slight decrease in EMV production at 18°C (Figure 7A). We further examined the morphology of EMVs by negative-staining TEM (Figures 7B,D) and cyro-EM (Figures 7C,E) and their size by DLS analysis (Supplementary Figure S2). The morphology of EMVs was similar between the mutants and the parent strain (Figures 4A,C,D, 7B–E). DLS analysis demonstrated that EMVs from the mutants showed a peak at 80–100 nm, which was identical to that of EMVs from the parent strain, whereas EMVs of larger size slightly increased for these mutants (Supplementary Figure S2). The result indicates that the lack of P49 and GspD2 did not affect the size of the majority of EMVs but altered their size distribution.

**FIGURE 8:**
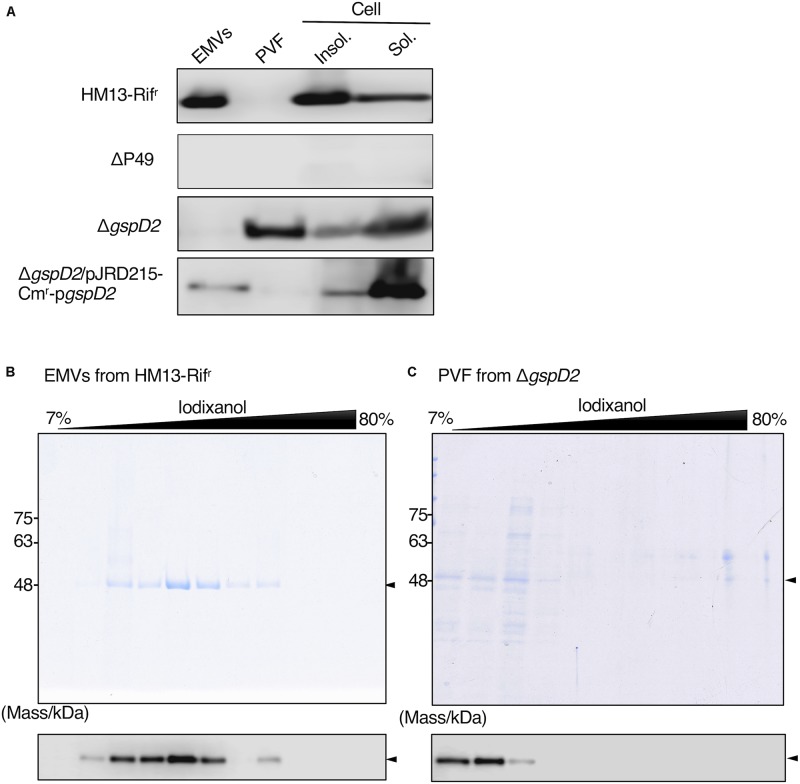
Effects of GspD2 depletion on the localization of P49. **(A)** Localization of P49 in HM13-Rif^r^, ΔP49, Δ*gspD2*, and Δ*gspD2*/pJRD215-Cm^r^-p*gspD2* strains of *S. vesiculosa* HM13 was analyzed by western blot analysis with an anti-P49 polyclonal antibody. The cells were grown in LB medium at 4°C to the stationary phase. EMVs and PVF were prepared from the culture supernatant by ultracentrifugation. Insoluble and soluble fractions of the cells were prepared by sonication of the cells and subsequent ultracentrifugation. Note that the film exposure conditions were different among different strains. Thus, the absolute amount of P49 cannot be compared among different strains based on its band intensity, whereas distribution patterns can be compared among them. **(B,C)** SDS-PAGE analysis of the fractions obtained by iodixanol density gradient ultracentrifugation of EMVs of HM13-Rif^r^ **(B)** and PVF of Δ*gspD2* **(C)**. P49 was detected by western blot analysis with the anti-P49 polyclonal antibody as shown in the lower panels. Arrowheads indicate the position of P49.

We then examined the potential role of GspD2 in the transport of P49 to EMVs. EMVs from Δ*gspD2* and HM13-Rif^r^ were isolated by ultracentrifugation. The soluble and insoluble fractions of the cells as well as the post-vesicle fraction (PVF; supernatant obtained after ultracentrifugation of the culture supernatant for the removal of EMVs as pellets) were collected as described in Section “Materials and Methods.” Analysis of each fraction of HM13-Rif^r^ by western blotting with an anti-P49 polyclonal antibody revealed the presence of P49 in EMVs (Figure 8A first panel). P49 was also detected in the cellular fractions, more abundantly in the insoluble fraction than in the soluble fraction. As a control experiment, we analyzed the P49 gene-disrupted mutant (ΔP49) and confirmed that the antibody was, indeed, specific to P49 (Figure 8A second panel). The disruption of *gspD2* remarkably affected the localization of P49. *gspD2* disruption resulted in the disappearance of P49 from EMVs, while it was detected in the PVF and cellular fractions (Figure 8A, third panel). The relative amounts of P49 in the PVF and soluble fractions were increased, while that in the insoluble fraction was decreased, in comparison with the distribution pattern of P49 in the HM13-Rif^r^. Complementation of Δ*gspD2* with an expression plasmid (pJRD215-Cm^r^-p*gspD2*) increased the relative level of P49 in EMVs while decreasing the relative amount in PVF (Figure 8A, fourth panel), indicating that GspD2 is involved in the transport of P49 to EMVs. It should be noted that the relative abundance of P49 in EMVs compared with that in the cellular fraction was lower for the complemented strain than for HM13-Rif^r^. A possible reason for this incomplete complementation will be described in Section “Discussion.”

To further investigate the localization of P49 in HM13-Rif^r^ and Δ*gspD2*, the EMV fraction of HM13-Rif^r^ and PVF of Δ*gspD2* were subjected to iodixanol density gradient centrifugation, and each fraction was analyzed by SDS-PAGE and western blotting with an anti-P49 antibody. P49 from HM13-Rif^r^ was enriched in the middle density fractions corresponding to 28–35% of iodixanol (Figure 8B). Similar to the results shown in Figure 2, we were able to confirm the presence of EMVs in these P49-enriched fractions by electron microscopy (data not shown). In the absence of GspD2, P49 was found in the low-density fractions, corresponding to 7–12% iodixanol, along with other proteins (Figure 8C), and the amount of P49 in these fractions was much lower than that recovered in the EMVs of HM13-Rif^r^ (Figures 8B,C). TEM analysis of these fractions indicated absence of EMVs (data not shown). These results together with those shown in Figure 8A suggest that GspD2 functions as an outer membrane channel that facilitates membrane association of P49 and its loading onto EMVs.

### Secretory Production of GFP as a Cargo of EMVs

**FIGURE 9:**
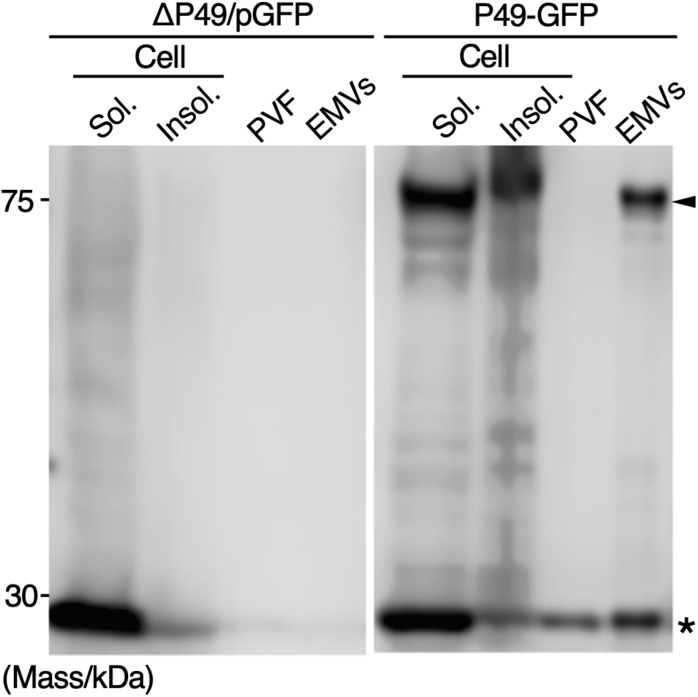
Secretory production of GFP as a cargo of EMVs. GFP fused to the C-terminus of P49 was expressed in *S. vesiculosa* HM13 (P49-GFP). As a control, GFP without being fused to P49 was expressed (ΔP49/pGFP). The cells were grown in LB medium at 18°C to the stationary phase. Distribution of the proteins in the insoluble fraction of the cells (Insol.), the soluble fraction of the cells (Sol.), PVF, and EMVs was analyzed by western blotting with anti-GFP antibody. The samples prepared from 100 μL culture were subjected to the analysis. Asterisk and arrowhead indicate the position of GFP and GFP fused to P49, respectively.

To examine whether secretory production of heterologous proteins as cargos of EMVs is possible, GFP fused to the C-terminus of P49 was expressed in *S. vesiculosa* HM13 (P49-GFP). As a control, GFP without being fused to P49 was expressed (ΔP49/pGFP). We found that GFP fused to P49 was transported to EMVs, whereas GFP by itself was not (Figure 9). Thus, it is possible to use P49 as a carrier to deliver the fusion partner to EMVs. GFP without being fused to P49 was also found in the P49-GFP strain. This is probably due to proteolytic cleavage of the fusion protein between P49 and GFP.

## Discussion

In this study, we have isolated a novel cold-adapted bacterium, *S. vesiculosa* HM13, which produces EMVs containing a single major cargo protein, P49. P49 was found to account for more than 90% of the total proteins of EMVs (Figure 2). This high relative abundance of a single cargo molecule in EMVs is very unusual compared with other bacterial EMVs (Lee et al., 2008). We have also found that this strain produces larger amounts of EMVs than a closely related strain of *Shewanella* and other Gram-negative mesophilic bacteria (Figure 3). Notably, the loss of P49 was not found to affect the production of EMVs remarkably. It is, therefore, possible to generate a large amount of EMVs that lack this major cargo protein using a P49-deficient mutant. Such “vacant” EMVs could be useful as a platform for the extracellular production of recombinant proteins, which can then be easily separated from cellular proteins by simple methods such as centrifugation or filtration. Considering these findings, *S. vesiculosa* HM13 can be regarded as a prospective host for the production of foreign proteins of high purity as a cargo of EMVs, and understanding of how the P49-containing EMVs are generated would facilitate such applications. Moreover, because *S. vesiculosa* HM13 is a cold-adapted bacterium, it would be useful as the host for the production of thermolabile proteins as a cargo of EMVs at low temperatures.

The biogenesis of EMVs in Gram-negative bacteria is known to occur by two different mechanisms. One mechanism involves budding and pinching off of EMVs from the outer membrane (Kulp and Kuehn, 2010; Schwechheimer and Kuehn, 2015; Roier et al., 2016). The other, recently proposed mechanism, involves explosive cell lysis, where shattered membrane fragments generated by cell lysis form EMVs (Turnbull et al., 2016). We found that the phospholipid composition of *S. vesiculosa* HM13 is different in the EMVs than in the cells, indicating that EMVs are produced through selective enrichment of a certain set of phospholipids (Figure 5). The great abundance of P49 in the EMVs implied the existence of a specific cargo transport mechanism for proteins as well. Indeed, as shown in Figure 2F, the outer membrane protein Omp74 is excluded from EMVs, indicating that selective cargo loading mechanism is operating for proteins. Overall, these results favor the former mechanism where EMVs are produced via blebbing and pinching off of the outer membrane rather than the latter mechanism involving cell lysis because selective loading of a certain set of cargo molecules is difficult when cell lysis occurs. FE-SEM analysis of the cell surface of this strain, showing many budding structures of which sizes were comparable to those of EMVs, also supports the budding mechanism of generation of EMVs (Figure 4E).

To obtain further insights into the mechanism of biogenesis of the P49-containing EMVs, we analyzed the genes in the vicinity of the P49 gene, expecting their involvement in this process. This analysis revealed genes coding for homologs of the T2SS machinery located upstream of the P49 gene, suggesting their involvement in P49 transport (Figure 6 and Table 1). Indeed, we found that the absence of GspD2, a protein that presumably constitutes a part of the T2SS-like machinery, resulted in mislocalization of P49 (Figure 8). It may be noticeable that the disruption of *gspD2* was not fully complemented by introduction of *gspD2*-expression plasmid. A possible reason for the incomplete complementation is that expression of the genes other than *gspD2* in the P49 gene-containing gene cluster may have been affected by the disruption of *gspD2*. Another possible reason is that expression of GspD2 from the plasmid was not sufficient to fully complement the loss of the genomic *gspD2* gene. Whatever the reason is, the complementation (although not full) indicates that GspD2 plays a role in the transport of P49 to EMVs.

**FIGURE 10:**
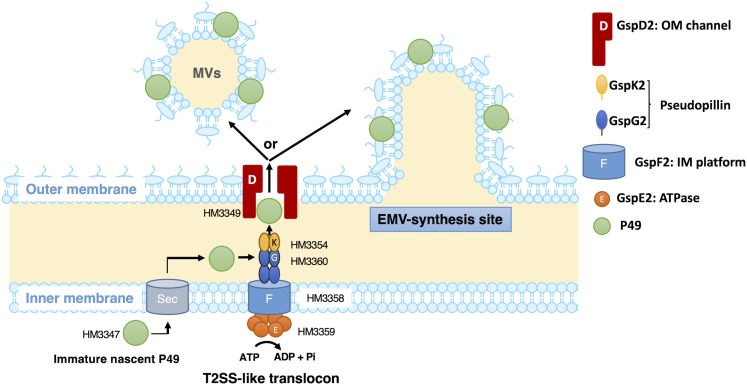
A possible model of the P49 secretion via the T2SS-like protein translocon of *S. vesiculosa* HM13. *S. vesiculosa* HM13 produces EMVs containing P49 as a major cargo protein. A newly synthesized P49 is translocated across the inner membrane probably by the Sec-translocon. Based on the predicted localization and function, GspE2 (HM3359), GspF2 (HM3358), GspG2 (HM3360), GspK2 (HM3354), and GspD2 (HM3349) are supposed to form the T2SS-like translocon. P49 in the periplasmic region is probably delivered to EMVs via the putative T2SS-like translocon. Targeting of P49 to EMVs may be ensured by specific interaction between P49 and EMVs (or their precursors budding from the cell surface) and/or between the T2SS-like system and EMVs (or their precursors). In the absence of the T2SS-like system, P49 is delivered to PVF, although inefficiently, via another secretion machinery. This may be carried out by the canonical T2SS (not depicted in this figure). The genes coding for GspCLM, GspHIJ, and GspN homologs are missing in the gene cluster containing the P49 gene (Figure 6). These components encoded by the canonical T2SS gene cluster or their counterparts may be incorporated into the T2SS-like machinery, which should be examined in future studies.

Based on the presence of a typical signal sequence at the N-terminus of P49 and the sequence similarity between the canonical T2SS and the putative T2SS-like machinery, it is plausible that P49 is first translocated from the cytoplasm to the periplasm by the Sec machinery with concomitant cleavage of the signal peptide and then delivered to EMVs through the T2SS-like machinery (Figure 10). Considering the potential role of GspD2 as an outer membrane channel and the predicted localization and function of GspE2 (HM3359), GspF2 (HM3358), GspG2 (HM3360), and GspK2 (HM3354) based on their sequence homologies with proteins of known function, it could be hypothesized that P49 is delivered to the outer membrane or the extracellular milieu by the T2SS-like machinery composed of these proteins. It would, hence, be a reasonable assumption that P49 delivery into EMVs requires specific interactions either between P49 and EMVs (or their precursors) or between the T2SS-like machinery and EMVs (or their precursors) or both. It is not clear yet if P49 is first released to the extracellular milieu and then becomes associated with EMVs, or if it is directly transferred from the T2SS-like machinery to EMVs without being released to the extracellular milieu. Because P49 is predicted to be a membrane protein, the former mechanism in which P49 is secreted without being embedded in the membrane may appear to be unlikely. However, we cannot exclude this mechanism, considering the peculiar nature of P49 that it can exist both in EMVs as a membrane-associated form and in PVF as a soluble form (Figure 8A). Future studies are necessary to clarify these details. In particular, it would be important to identify interaction partners of P49 and the T2SS-like machinery. Topological analysis and 3D-structural analysis of P49 should be performed to elucidate the structural basis underlying the above-mentioned peculiar nature of P49. It is also important to examine, for example, by gene deletion experiments whether GspCLM, GspHIJ, and GspN of the canonical T2SS are used as components of the T2SS-like machinery or their counterparts take over their roles in this machinery.

It was previously reported that a *Vibrio cholerae* protease, PrtV, is secreted via canonical T2SS in association with EMVs (Rompikuntal et al., 2015). In contrast to this case, *S. vesiculosa* HM13 has T2SS-like machinery besides the canonical T2SS (Figure 6 and Tables 1, 2), and the T2SS-like machinery is required for the transport of P49 to EMVs. In the absence of the T2SS-like machinery, P49 was found to accumulate in the soluble fraction of the cells and was partly delivered to PVF instead of EMVs (Figure 8A), although the levels of P49 found in the PVF in this mutant were much lower than that found in EMVs of HM13-Rif^r^ (Figures 8B,C). Thus, as for P49, the canonical T2SS does not function as a cargo delivery system for EMVs, although it may inefficiently function as a secretion system to deliver P49 to PVF as discussed below.

It is notable that P49 was recovered in the soluble fraction (the soluble cellular fraction and PVF) in the *gspD2*-disrupted mutant (Figure 8A), although prediction of membrane-spanning regions of P49 suggested the presence of two transmembrane segments and P49 was indeed found to be localized in the insoluble cellular fraction in HM13-Rif^r^ cells (Figure 8A). These results suggest that P49 adopts both membrane-associated and non-membrane-associated forms. Such dual forms have been reported for other proteins such as the C-terminal domain of OmpA, a major outer membrane porin of *E. coli* (Wang, 2002). OmpA adopts a 16-stranded ß-barrel conformation and an eight-stranded ß-barrel conformation (Smith et al., 2007). In the former conformation, the C-terminal domain is integrated into the outer membrane, whereas in the latter conformation, it is in the periplasmic space. In order to understand the mechanism underlying the dual localizations of P49, it is necessary to clarify how P49 interacts with EMVs in more detail and whether there is a conformational difference between P49 of the wild-type cells and that of the mutant.

We found that P49 was delivered to the extracellular milieu by the *gspD2*-disrupted mutant (Figure 8A) although the amount of P49 secreted by this mutant was much lower than that by the parent strain (Figures 8B,C). Thus, we have to assume that P49 was secreted inefficiently by the function of protein secretion machinery that does not require GspD2 in this mutant. We speculate that the canonical T2SS may be involved in this process although the canonical T2SS by itself does not deliver P49 to EMVs as we described above. A possible interpretation of this result is that there is a conformational difference between P49 delivered through the canonical T2SS and that through the T2SS-like machinery. Alternatively, there may be an interaction between the T2SS-like machinery and EMVs (or their precursors) to directly deliver P49 to EMVs, whereas such interaction is missing for the canonical T2SS.

Although the detailed mechanism of cargo transport and biogenesis of EMVs remains to be elucidated, the results obtained in the present study revealed a new pathway for cargo transport and delivery to EMVs. This study also introduces a novel platform for the development of extracellular protein production systems. Indeed, we found that a foreign protein (GFP) can be delivered to EMVs by being fused to P49 (Figure 9). It should be noted here that P49-free GFP was detected in EMVs in addition to P49-fused GFP probably because of proteolytic cleavage of the fusion protein after being transported to EMVs. Regulation of this putative proteolytic activity is supposed to be important to increase the utility of this system. It may be possible to obtain P49-free foreign protein by facilitating the cleavage, whereas it may be possible to obtain the fusion protein with higher purity by suppressing the cleavage. To further understand EMV biogenesis and to broaden the range of applications of EMVs, studies are underway for the mechanistic analysis of cargo transport and EMV biogenesis; improvement of the systems for extracellular production of proteins as cargoes of EMVs using *S. vesiculosa* HM13 is also in progress.

## Data Availability Statement

The datasets generated for this study can be found in the DDBJ: LC460999, LC461000, LC431043, LC431042, LC431041, LC431040, LC431039, LC431038, LC431037, LC431036, LC431035, LC431034, LC431033, LC431032, LC431031, LC431030, LC431029, LC431028, LC431027, LC431026, LC431025, LC431024, LC431023, LC431022, LC431021, LC431044, LC431045, LC431046, LC431047, LC431048, LC431049, LC431050, LC431051, LC431052, LC431053, LC431054, LC431055. Newly determined nucleotide sequence data have been deposited to DDBJ. The accession numbers of the sequence data for 16S rRNA genes of *S. vesiculosa* HM13 are LC460999 and LC461000, and the accession numbers of other sequence data are shown in Tables 1, 2.

## Author Contributions

CC, JK, and TK designed the study. SK isolated *S. vesiculosa* HM13. AT, CK, and TI contributed to electron microscopic analysis. CC performed the experiments. CC, JK, and TK discussed the results and wrote the manuscript.

## Conflict of Interest

AT was employed by the company Marine Works Japan, Ltd. The remaining authors declare that the research was conducted in the absence of any commercial or financial relationships that could be construed as a potential conflict of interest.

## Footnotes

1 http://enve-omics.ce.gatech.edu/ani/index

2 http://rast.nmpdr.org

3 https://blast.ncbi.nlm.nih.gov/Blast.cgi

4 https://embnet.vital-it.ch/software/TMPRED_form.html

5 http://harrier.nagahama-i-bio.ac.jp/sosui/sosuigramn/sosuigramn_submit.html
