# Supplementary material for: Isolation of a Novel Bacterial Strain Capable of Producing Abundant Extracellular Membrane Vesicles Carrying a Single Major Cargo Protein and Analysis of Its Transport Mechanism
Source: Front Microbiol. 2020 Jan 14;10:3001. doi: 10.3389/fmicb.2019.03001 (PMC6971210; doi:10.3389/fmicb.2019.03001)
Supplement: Supplementary file 1 [file Data_Sheet_1.pdf]

Table S1 Primers used in this study

Primers	Description	Sequence
16S-fwd	Amplification of 16S rDNA	5’-AGAGTTTGATCMTGGCTCAG-3’ M(A/C)
16S-rev		5’-TACGGYTACCCTTGTTACGTTACGACTT-3’ Y(C/T)
pKNOCK-fwd	Preparation of linearized pKNOCK-Km^r	5’-GGGCTGCAGGAATTC-3’
pKNOCK-rev		5’-GGGGGATCCACTAGT-3’
P49-fwd	P49 gene disruption	5’-ACTAGTGGATCCCCCGTAGTCCCAACTATCGCAGT-3’
P49-rev		5’-GAATTCCTGCAGCCCTACCGATTACGCCTAGGTCAT-3’
gspD2-fwd2	GspD2 gene disruption	5’-ACTAGTGGATCCCCCGTTGAGGCTACATCGACTAT-3’
gspD2-rev2		5’-GAATTCCTGCAGCCCTTGGGCACTTCGTAAATGT-3’
pJRD215-fwd	Preparation of linearized pJRD215	5’-TAGTATAGTCTATAGTCCGTGG-3’
pJRD215-rev		5’-CGTAATCCATGGATCAAGAG-3’
up1000-fwd	*gspD2* complementation	5’-CTCTTGATCCATGGATTACGTTATCAACAAGCCCTGAAGCATCACTTCA-3’
up1000-rev		5’-AATAGGTTGTTATTAGCCAAACCATCTCCAATTTCATGAAATTTGGACT-3’
gspD2-fwd	*gspD2* complementation	5’-TTGGCTAATAACAACCTATTTACCCGCG-3’
gspD2-rev		5’-ACGGACTATAGACTATACTATTAGTCCTGCTGCAGATCAATATTATTGAG-3’
up500-fwd	ΔP49/pGFP construction	5’-ACTAGTGGATCCCCCGTTTTTGCAGGCTAGAAGTCAAAACTTTGT-3’
up500-rev		5’-TTCTCCTTTACTCATACCATCTCCAATTTCATGAAAT-3’
pGFP-fwd	ΔP49/pGFP construction	5’-ATGAGTAAAGGAGAAGAACTTTTCACTGGA-3’
pGFP-rev		5’-GAATTCCTGCAGCCCCTATTTGTATAGTTCATCCATGCCATGTGT-3’
P49C-fwd	P49-GFP construction	5’-ACTAGTGGATCCCCCTCCTACAGGTACAACAGTACCAAACGAT-3’
P49C-rev		5’-TTCTCCTTTACTCATCTTAGAACCGTTAGAAGTGTTAGATACGAATCCA-3’
GFP-fwd	P49-GFP construction	5’-ATGAGTAAAGGAGAAGAACTTTTCACTGG-3’
GFP-rev		5’-GAATTCCTGCAGCCCCTATTTGTATAGTTCATCCATGCCATGTG-3’
pJRD215 mluI-fwd	Confirmation of introduction of the pJRD215-derived plasmids	5’-CCTGTCTCTTGATCCATGGATTACG-3’
pJRD215 speI-rev		5’-ATTCCACGGACTATAGACTATACTA-3’
pKNOCK check-rev	Confirmation of the GFP gene insertion into the genome	5’-CCTCGAGGTCGACGGTATCG-3’

Table S2 Strains and plasmids

Strain	Description	Reference
HM13	*S. vesiculosa* HM13 wild type (JCM 33296), the parent strain of HM13-Rif^{r}	This study
HM13-Rif^{r}	Rifampin-resistant mutant of *S. vesiculosa* HM13, the parent strain of ΔP49, Δ*gspD2*, and P49-GFP	This study
ΔP49	P49 gene (HM3347)::pKNOCK-Km^{r}	This study
Δ*gspD2*	*gspD2* (HM3349)::pKNOCK-Km^{r}	This study
ΔP49/pGFP	ΔP49 strain harboring pJRD215-Cm^{r}-pGFP	This study
P49-GFP	Recombinant HM13-Rif^{r} strain that produces GFP fused to the C-terminus of P49	This study
Shewanella livingstonensis Ac10	A cold-adapted *Shewanella* strain, a control for EMV productivity analysis	(Kulakova et al., 1999)
Shewanella oneidensis MR-1	A mesophilic *Shewanella* strain, a control for EMV productivity analysis	(Myers and Nealson, 1988)
Pseudomonas putida KT2440	A control strain for EMV productivity analysis	(Bagdasarian et al., 1981)
Escherichia coli MG1655	A control strain for EMV productivity analysis	(Guyer et al., 1981)
Escherichia coli S17-1/ *λ pir*	A donor strain for conjugative transformation, S17-1 derivative, host for *pir*–dependent plasmids	(Simon et al., 1983)
**Plasmid**	**Description**	**Reference**
pJRD215-Cm^{r}	A broad-host-range vector	(Toyotake et al., 2018)
pKNOCK-Km^{r}	A gene knockout plasmid, RP4 *oriT* and R6K-*ori*, Km^{r}	(Alexeyev, 1999)
pJRD215-Cm^{r}-p*gspD2*	An expression plasmid coding for *gspD2*	This study
pJRD215-Cm^{r}-pGFP	An expression plasmid harboring the GFP gene fused with the upstream flanking region of the P49 gene	This study
pGreen	A plasmid used as a template for PCR amplification of the GFP gene	(Hellens et al., 2000)

Materials and Methods

Membrane fractionation, western blot and dot blot analyses, and enzyme assay

The cells of *S. vesiculosa* HM13 were grown in LB medium at 18 °C to the stationary phase and were harvested from 4 mL culture by centrifuged at 6,800 × *g* for 10 min at 4 °C. Harvested cells were suspended in DPBSS (4 mL) and disrupted by sonication. Low-speed centrifugation (8,000 × *g*) was performed at 4 °C for 15 min to remove undisrupted cells. The membrane fraction was pelleted from the supernatant by ultracentrifugation for 1 h at 270,000 × *g* at 4 °C. Membrane fractions were suspended in 300 μl DPBSS, placed on top of a three-step sucrose gradient (0.77 M, 1.44 M, and 2.02 M sucrose (1.5 mL each)), and separated by ultracentrifugation for 16 h at 230,000 × *g* at 4 °C. Fractions (0.4 mL each) were collected from the top and subjected to SDS–PAGE. The protein samples were separated on a 5-20% SuperSep™ Ace SDS-PAGE gel (Wako Pure Chemical Industries) and blotted onto a PVDF membrane (Merck Millipore Co.).

P49 was detected by using polyclonal anti-P49 antibody prepared as described in Materials and Methods of the main text. The antiserum was diluted to 1:50,000. Omp74 was detected with previously prepared polyclonal rabbit antiserum raised against Omp74 of *S. livingstonensis* Ac10 (Dai et al., 2012). The antiserum was diluted to 1:40,000. Lypopolysaccharides (LPS) were detected with 10,000-fold diluted anti-LPS antibody purchased from LSBio (Seattle, WA) by dot blotting. Blotting Grade Affinity Purified Goat Anti-Rabbit IgG (H+L)-HRP Conjugate (Bio-Rad Laboratories, Inc., Hercules, CA) was used as a secondary antibody at a final dilution of 1:50,000. The immunoblot detection was performed using the Chemi-Lumi One Ultra (Nacalai Tesque, Kyoto, Japan) and a C-Digit Blot Scanner (LI-COR Biosciences, Lincoln, NE).

To determine the inner membrane fractions, an enzyme activity of NADH oxidase, an inner membrane marker enzyme, was assessed. NADH (0.2 mM, 10 μl) was mixed with NADH oxidase samples (100 μl) in a 96-well microplate. The plate was incubated at room temperature for 30 min. The decrease of NADH was measured spectrophotometrically at 340 nm to determine the activity of NADH oxidase.

Dynamic light scattering analysis

The EMVs produced by the parent strain (HM13-Rif^r) grown at 4 °C and 18 °C and those produced by ΔP49 and Δ*gspD2* grown at 18 °C were subjected to dynamic light scattering (DLS) analysis. Isolated EMVs were suspended in 0.1 μm-filtered 500 μl DPBS (Dulbecco's PBS) and subjected to Intensity-weighted GAUSSIAN Analysis (Vesicles model) with Nicomp Model 370 Submicron Particle sizer (Particle Sizing Systems, Santa Barbara, CA).

Results

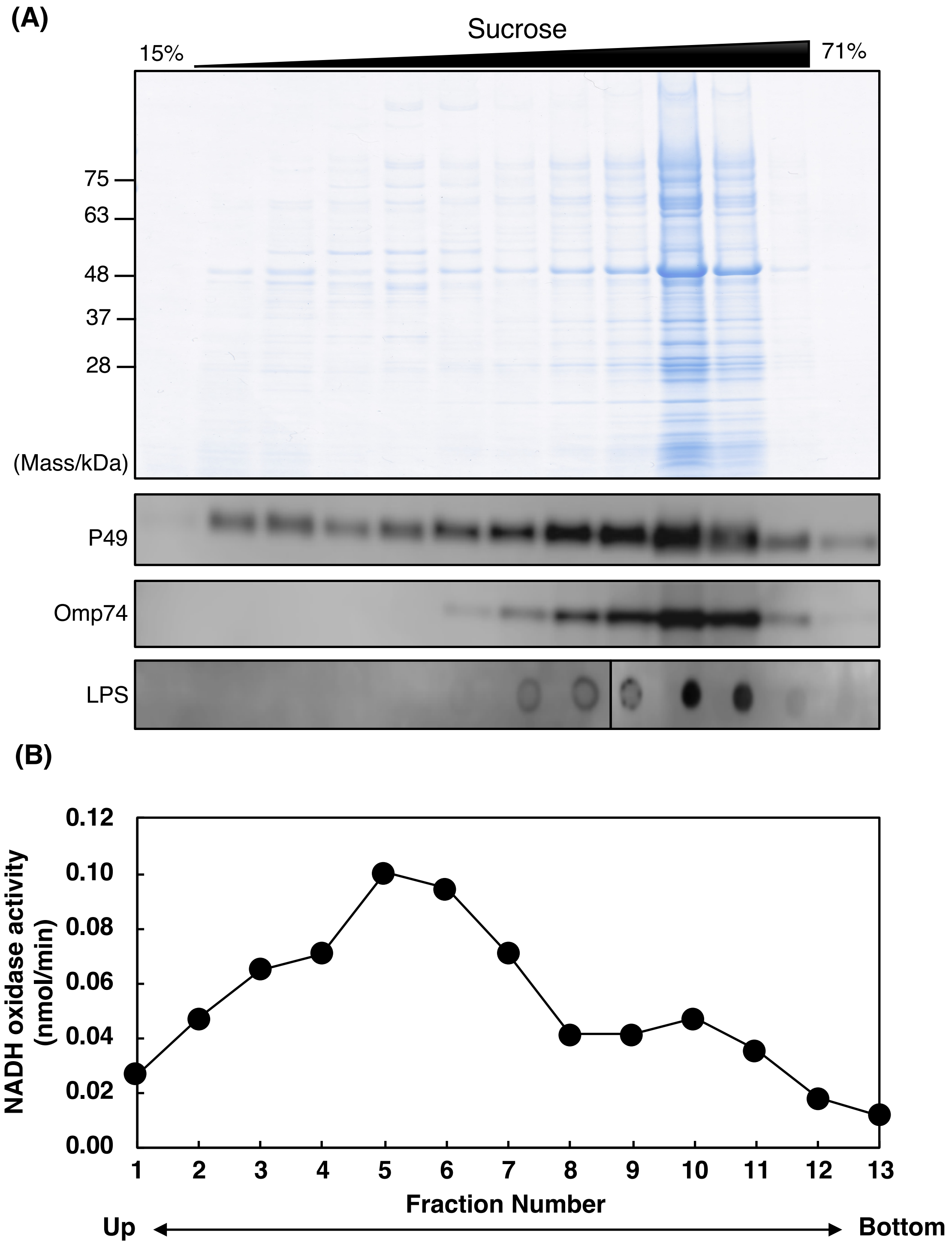

Figure S1 Membrane fractionation to determine the localization of P49

(A) SDS-PAGE and western blot analysis of the fractions obtained by sucrose density gradient ultracentrifugation of the cellular membrane fraction. Localization of P49

and Omp74 were analyzed by western blot analysis with an anti-P49 polyclonal antibody and an anti-Omp74 antibody, respectively. LPS, a marker of the outer membrane, was analyzed by dot blotting with an anti-LPS antibody. (B) The activity of NADH oxidase, a marker enzyme of the inner membrane, in each fraction.

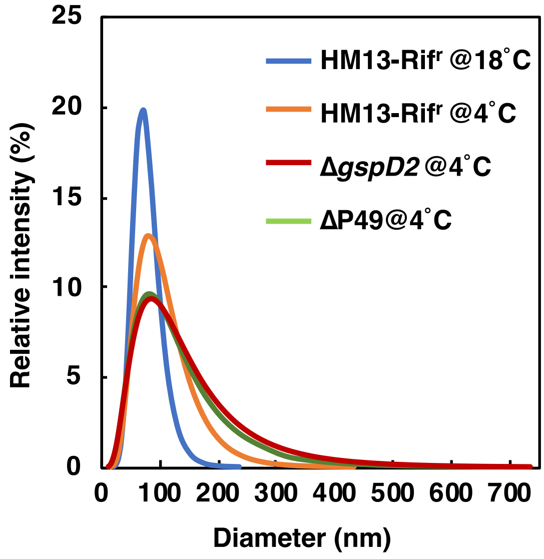

Figure S2 Size distribution of EMVs

HM13-Rif^r was grown at 4 °C and 18 °C, and ΔP49 and Δ*gspD2* were grown at 4 °C. Size distributions of EMVs from HM13-Rif^r grown at 4 °C (orange line) and 18 °C (blue line), ΔP49 (green line), and Δ*gspD2* (red line) were measured with Nicomp Model 370 Submicron Particle Sizer.

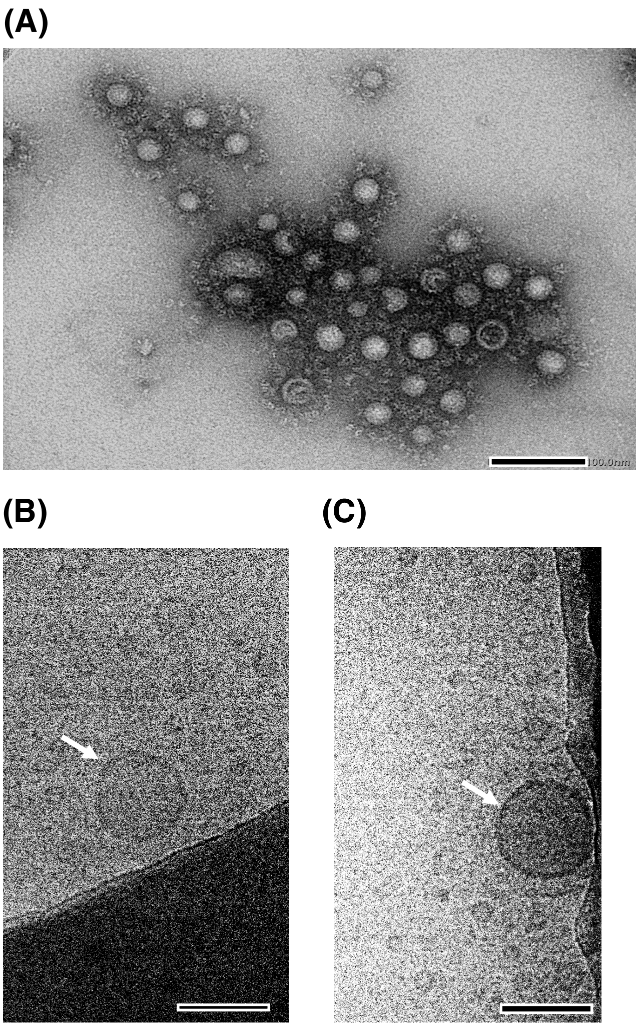

Figure S3 Electron microscopic images of EMVs of *S. vesiculosa* HM13

Negative-stained TEM (A) and cryo-EM (B and C) images of EMVs from *S. vesiculosa* HM13 grown at 18 °C. Arrows indicate EMVs surrounded by a high electron density region. Bars represent 100 nm.

References

1. Alexeyev, M. F. (1999). The pKNOCK series of broad-host-range mobilizable suicide vectors for gene knockout and targeted DNA insertion into the chromosome of Gram-negative bacteria. *Biotechniques* 26:824–826.
2. Bagdasarian, M., Lurz, R., Riickert, B., Bagdasarian, M. M., Frey, J., Timmis, K. N. (1981). Specific-purpose plasmid cloning vectors. II. Broad host range, high copy number, RSF1010-derived vectors, and a host-vector system for gene cloning in *Pseudomonas*. *Gene* 16:237–247.
3. Dai, X. Z., Kawamoto, J., Sato, S. B., Esaki, N., Kurihara, T. (2012). Eicosapentaenoic acid facilitates the folding of an outer membrane protein of the psychrotrophic bacterium, *Shewanella livingstonensis* Ac10. *Biochem Biophys Res Commun* 425:363–367.
4. Guyer, M. S., Reed. R. R., Steitz, J. A., Low, K. B. (1981). Identification of a sex-factor-affinity site in *E. coli* as gamma delta. *Cold Spring Harb Symp Quant Biol* 45:135–40.
5. Hellens, R. P., Edwards, E. A., Leyland, N. R., Bean, S., Philip, M. (2000). pGreen : a versatile and flexible binary Ti vector for *Agrobacterium*-mediated plant transformation. *Plant Mol Biol* 42:819–832.
6. Kulakova, L., Galkin, A., Kurihara, T., Yoshimura, T., Esaki, N. (1999). Cold-active serine alkaline protease from the psychrotrophic bacterium *Shewanella* strain Ac10: Gene cloning and enzyme purification and characterization. *Appl Environ Microbiol* 65:611–617.
7. Myers, C. R., Nealson, K. H. (1988). Bacterial manganese reduction and growth with manganese oxide as the sole electron acceptor. *Science* 240:1319–1321.
8. Simon, R., Priefer, U., Pühler, A. (1983). A broad host range mobilization system for *in vivo* genetic engineering: transposon mutagenesis in Gram negative bacteria. *Bio Technol* 1:784–791.
9. Toyotake, Y., Cho, H. N., Kawamoto, J., Kurihara, T. (2018). A novel 1-acyl-*sn*-glycerol-3-phosphate *O*-acyltransferase homolog for the synthesis of membrane phospholipids with a branched-chain fatty acyl group in *Shewanella livingstonensis* Ac10. *Biochem Biophys Res Commun* 500:704–709.
